# Polypharmacy and Potentially Inappropriate Medications in Patients With Dementia

**DOI:** 10.1093/geroni/igaa057.673

**Published:** 2020-12-16

**Authors:** Ryo Nonaka, Atsuhiro Kanno, Takahiro Ohara, Kazuhiro Sumitomo, Shigeru Sato, Isabelle Miyazawa, Nobuo Koinuma, Katsutoshi Furukawa

**Affiliations:** Tohoku Medical and Pharmaceutical University, Sendai, Japan

## Abstract

INTRODUCTION: The use of polypharmacy and potentially inappropriate medication (PIM) is a critical issue in geriatrics. Furthermore, the number of patients with dementia is dramatically increasing worldwide. In this study, we investigated: (1) if the states of polypharmacy and PIM differed in patients with and without dementia; (2) the types of medicine that were commonly prescribed; (3) the types of dementia that resulted in the prescription of multiple medicines; (4) if there was a correlation between the number of medicines and the number of medical institutions (hospitals and clinics) that the patients attended. METHODS: In total, 216 patients who were 65 years of age and older were analyzed. The number of medicines prescribed and the medical institutions they attended were counted through the electronic medical charts of Tohoku Medical and Pharmaceutical University Hospital. We employed the Beers 2019 criteria for the definition of PIM. RESULTS: (1) The number of prescribed medicines was not significantly different between patients with dementia and those without dementia. (2) Anti-hypertensives and gastro-intestinal medicines were the most commonly prescribed medicines in patients with and without dementia. (3) Patients with “dementia with Lewy bodies” and “mixed dementia” were prescribed the highest number of medicines. (4) The number of medicines and PIM use were significantly and positively correlated with the number of medical institutions. CONCLUSIONS: The number of medical institutions strongly affected the number of medicines prescribed and PIM use. Efforts should be made to organize and reduce the number of medical institutions that a patient attends.

